# Strategic Location-Based Random Routing for Source Location Privacy in Wireless Sensor Networks

**DOI:** 10.3390/s18072291

**Published:** 2018-07-15

**Authors:** Lilian C. Mutalemwa, Seokjoo Shin

**Affiliations:** Department of Computer Engineering, Chosun University, Gwangju 61452, Korea; lilian.mutalemwa@gmail.com

**Keywords:** source location privacy, wireless sensor network, adversary, random routing, diversion node, back tracing

## Abstract

Wireless sensor networks (WSNs) are deployed in sensitive applications, such as in military and asset monitoring. In these applications, it is important to ensure good source location privacy. This is owing to the open nature of WSNs and the easiness of an adversary to eavesdrop on sensor communication and back trace the location of the source node. This paper proposes a scheme to preserve the source location privacy based on random routing techniques. To achieve high privacy, packets are randomly routed from the source to the sink node through strategically positioned mediate or diversion nodes. The random selection of mediate or diversion nodes is location-based. Depending on the location of the source node, packets are forwarded through different regions of the network. The proposed scheme guarantees that successive packets are routed through very different routing paths and adversaries find it confusing to back trace them to the source node location. Simulation results demonstrate that the proposed scheme effectively confuses the adversary and provides higher source location privacy to outperform other routing-based source location privacy schemes.

## 1. Introduction

A wireless sensor network (WSN) is a network which consists of spatially distributed autonomous sensors with the aim of monitoring various physical and environmental conditions including asset monitoring and tracking [1]. A recent implementation of an asset monitoring network is the Wildlife Crime Technology report which is based on a WSN that is used to monitor a large area where animals roam [1,2]. In monitoring applications, nodes operate by monitoring their surroundings to detect the presence of an asset. When the asset is detected, the node which detects the asset becomes a source node and transmits a message to the sink to report the presence of the asset in its surroundings [2]. Often, the distance between the source node and sink is longer than the transmission range of sensor nodes, making multi-hop communication a viable mode of transmission [1].

One of the challenges that face multi-hop communication wireless networks is creating secure and private applications. This is owing to their potential to expose important information as packets are broadcasted across the network. Security measures, such as cryptographic techniques like encryption, decryption, and authentication, are used to secure the integrity of data and protect the content of the packets but the context of the broadcast remains exposed to adversaries. Adversaries can use expensive radio transceivers to interact with the network, monitor the pattern of broadcasts and back trace them to the location of the source node [3]. This has motivated researchers to design schemes for preserving source location privacy in WSNs.

**Figure 1:**
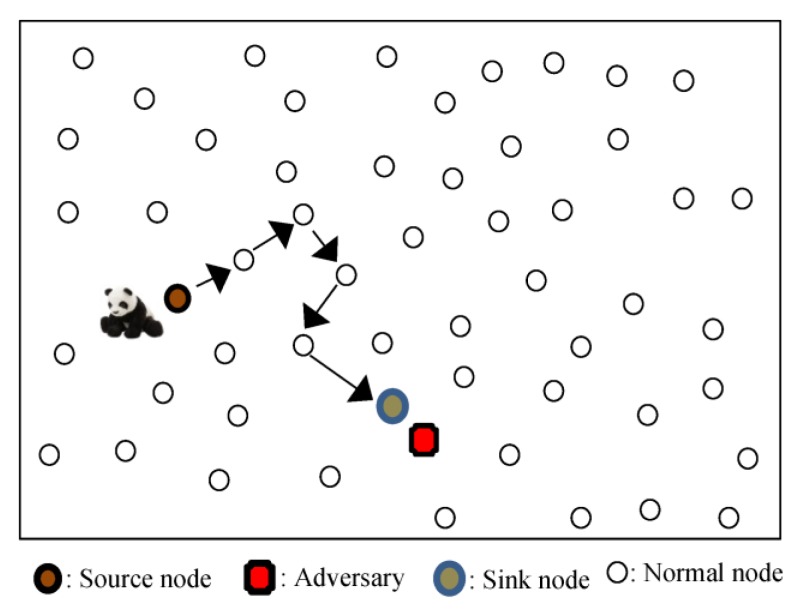
Example source node, sink node, and adversary configuration in a WSN.

Privacy can be defined as a guarantee that information can only be observed or deciphered by those that it is intended for [1]. Source location privacy preservation is the process of keeping the location of a source node hidden from adversaries in an asset monitoring network [1]. Figure 1 shows an example asset monitoring network where the monitored asset is a panda. Using WSNs to monitor endangered giant pandas in a bamboo forest is a common example of WSN monitoring applications. Pandas are high-value assets that need protection. In 2003, a single piece of panda fur was sold in Chongqing, China for $66,500 [4]. To protect the pandas from hunters in the forest, each panda will have an electronic tag which emits a signal for detection by the sensor nodes in the network. In the scenario presented in Figure 1, the adversary is initially located in the vicinity of a sink node to enable it to sense packets arriving at the sink node for back tracing attacks. Adversaries who start at sink node locations guarantee that they receive packets since the sink node is the destination for all the packets. The objective of a source location privacy scheme is to make the back tracing of the location of a source node a complex task for the adversary.

There exist numerous routing-based privacy preservation schemes to address the issue of source location privacy in WSNs. These schemes use routing strategies that prevent the adversaries from tracing the source node location through traffic monitoring and analysis. They aim to deliver each packet at the sink node through different paths which improve privacy by making it difficult for adversaries to guess the route for the next packet. This paper addresses the issue of source location privacy in WSNs by proposing a routing scheme with better privacy performance compared to three existing schemes: (1) shortest path routing [5]; (2) phantom single-path routing [6]; (3) directional random routing [7]. A common limitation for these three schemes is that they provide poor privacy for source nodes located near the sink [8]. In the shortest path routing scheme, the packet forwarding algorithm employs the single shortest path from the source node to the sink node to make it easy for the adversary to trace it back to the source node. In phantom single-path routing and directional random routing, the selection of the next-hop node always obeys a constant rule which leads to short routing paths for source nodes in near-sink regions. Additionally, if the packets are from the same source node in the near-sink region, the routing paths become strongly related to each other and get closer and closer to each other, thus leading to poor privacy [8,9].

**Figure 2:**
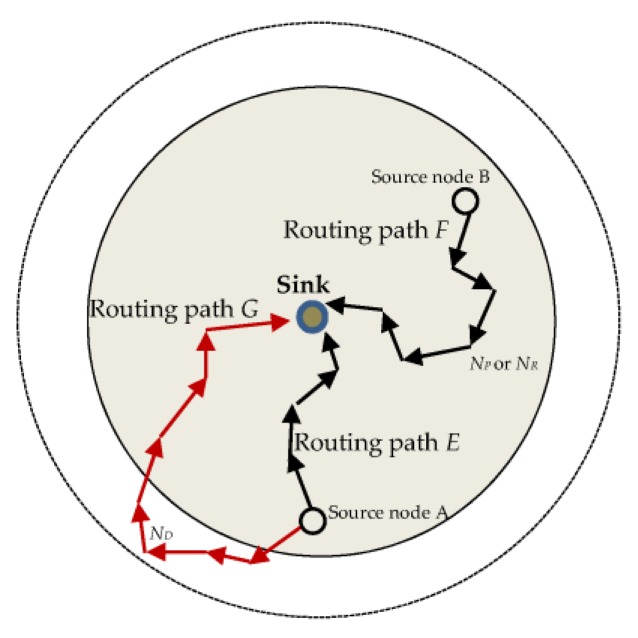
Routing paths for shortest path routing (path *E*), phantom single-path routing, or directional random routing (path *F*), and for the proposed routing scheme (path *G*).

Figure 2 demonstrates the limitations of shortest path routing, phantom single-path routing, and directional random routing. It also demonstrates the routing strategy of the proposed scheme for source nodes in near-sink regions. Routing path *E* uses the shortest path routing which delivers packets to the sink through the shortest route, thus causing poor privacy. The routing path *F* can either use phantom single-path routing, or directional random routing, which delivers packets to the sink through either the phantom node *N_P_* or the reference node *N_R_*. The routing path *F* results in short and strongly related routing paths, which ultimately leads to poor privacy for source nodes in near-sink regions. The routing path *G* uses the proposed scheme. It delivers packets to the sink Trough diversion node *N_D_* which is located away from the near-sink region. The routing path *G* delivers packets to the sink through a longer and more random routing path which improves the source location privacy as compared to routing paths *E* and *F*. It is more confusing for an adversary to back trace the routing path *G* than it is for routing paths *E* and *F*.

In this paper, a routing scheme with strategically positioned diversion and mediate nodes is proposed to provide a high level of source location privacy. The routing of packets is location-based. Nodes located in a near-sink region route their packets through randomly selected diversion nodes, while nodes located away from the sink region route their packets through randomly selected mediate nodes. Diversion and mediate nodes are strategically positioned to guarantee that successive packets use different routes to arrive at the sink. If an adversary decides to trace back the routes to the source node, it finds multiple possible routes through randomly selected diversion or mediate nodes, thus making it difficult to guess the route for the next packet. The scheme operates in two phases. In phase 1, packets from a source node are randomly forwarded to a random diversion or mediate node. In phase 2, diversion or mediate nodes randomly forward packets to the sink node using random walk routing.

The main contributions of this paper can be summarized as follows: (1) to address the limitations of shortest path routing, phantom single-path routing and directional random routing schemes by proposing a new routing scheme that uses strategically positioned mediate and diversion nodes to significantly improve path diversity of the routing paths between the source and sink nodes; (2) to conduct a series of experiments to evaluate the performance of the proposed routing scheme; (3) to demonstrate that the proposed scheme provides stronger source location privacy than shortest path routing, phantom single-path routing, and directional random routing schemes.

The remainder of this paper is organized as follows: Section 2 presents a review of the literature on routing schemes for source location privacy. Section 3 gives overview of the system including the network and adversary models. The proposed strategic location-based random routing scheme is described in details in Section 4. Privacy analysis of the proposed routing scheme is presented in Section 5. Performance analysis and evaluation together with the simulation results are presented in Section 6. The paper concludes in Section 7.

## 2. Related Work

Since the problem of source location privacy was put forward in [5] using the Panda-Hunter game, several research studies have proposed routing schemes to preserve source location privacy in WSNs. This section highlights the features of a few schemes. This work categorizes the routing schemes into fake source routing, phantom and random walk routing, intermediate node routing, tree routing, and angle-based routing.

The first fake source routing-based scheme was introduced in [5]. The scheme uses a set of fake source nodes to act as real sources. The fake sources generate packets to model the network traffic in a way that confuses an adversary by diverting attention away from the real source. Fake packets are of the same length as the real packets, and they are encrypted so as to make it difficult for adversaries to tell the difference between fake and real packets. Several versions of fake source routing exist, including short-lived fake source routing, persistent fake source routing, dynamic fake source routing, and a distributed solution that combines fake source routing and phantom routing [10]. Fake source routing schemes are often criticized for their poor privacy and high-energy consumption. The most significant limitation of fake source routing schemes is the increased volume of packets required to be broadcasted in order to provide efficient source location privacy. The high volume of packets in the network leads to high-energy consumption and to an increased number of collisions, thus resulting in a reduced packet delivery ratio.

Phantom routing and random walk routing were introduced in [5]. The schemes were proposed to improve the limitations of fake source routing scheme. Packet forwarding in phantom routing involves two phases: (1) random walk to an arbitrary node referred to as a phantom source, and (2) a succeeding flooding or single-path routing. Phantom single-path routing (PSRS) [6] is more efficient than phantom flooding [5] in terms of the safety period and energy consumption. Limitations of phantom source routing include lesser privacy, especially for source nodes located near the sink node. Several versions of phantom routing exist, including directed walk PSRS [5] and the greedy random walk (GROW) [11]. GROW uses two-way random walks, one from the source node and another from the sink node. The two random walks meet at a randomly selected phantom node. The phantom node then uses the path established by the random walk from the sink to the phantom node to route the packet from the source node to the sink. GROW achieves increased privacy by relaxing the requirement for the delivery latency.

An intermediate node-based routing scheme was introduced in [3]. The scheme randomly selects an intermediate node using a predefined constrained region around the source node. The scheme operates in two phases: (1) the source node forwards the packet to an intermediate node located at a determined minimum distance from the source; (2) the intermediate node routes the received packet to the sink node. The scheme provides better privacy than phantom routing but with higher energy consumption. Similar to phantom routing, this scheme provides poor privacy for source nodes near the sink. The routing paths get shorter as the source node gets closer to the sink node. A three-phase intermediate node-based routing scheme was proposed in [12]. In the first phase, the source node randomly selects an intermediate node in the network and then forwards the packet to the randomly selected intermediate node before it is routed to a ring node. In the second phase, the data packet is mixed with other packets through a network mixing ring. In the third phase, the packet is forwarded to the sink node from nodes in the mixing ring. The main limitation of this scheme is that energy consumption in the network is unbalanced. Ring nodes are more likely to drain their batteries faster than other nodes. Moreover, the sink node is surrounded by ring nodes which imply that if the ring nodes drain all their energy, sink nodes may become isolated from the nodes outside the ring.

The tree-based diversionary routing scheme [8] operates in two phases. It establishes the direct backbone route path to the network edge based on phantom routes for establishing homogeneous trees. Subsequently, it establishes many redundant diversionary routes in non-hotspot regions with abundant energy. It works by letting each node create its own root path that extends to the network border. The root path helps divert the attention of adversary from the real packet route. It establishes a phantom node away from the source node and then establishes a tree routing path towards the sink with strategically created diversionary routes as its branches. Fake source nodes are placed at the end of the diversionary routes to confuse the adversary. The main limitation for this scheme is that it uses considerable energy for each node to create their root paths that extend to the end of the network border. Although the path diverts the adversary from real paths and increases the privacy level, the energy consumption is too high.

The angle-based dynamic routing scheme [13] uses the location information of the nodes and calculates two inclination angles formed between nodes: (1) the inclination angle between a forwarding node and a receiving node; (2) the inclination angle between the forwarding node and the sink node. The angles are used to form a candidate set of neighboring nodes to forward the packet. One of the nodes in the candidate set is selected randomly as the next-hop node. The candidate set changes at every packet forwarding instance to form multiple paths towards the sink node for high privacy level. Other angle-based routing schemes include two-phantom angle-based routing [14] and constrained random routing [15]. Two-phantom angle-based routing scheme considers a triplet for selecting the phantom nodes. A triplet is a group of three nodes formed on the basis of three parameters: (1) their distance from the sink node; (2) their location information; (3) the inclination angle between them. Phantom selection is performed for every packet forwarding instance, thus creating multiple paths for the packets. The routing path for the packets changes dynamically, thereby increasing the safety period without a significant increase in the packet delivery latency. Constrained random routing is based on the transmission of offset angles and constrained probability. To prevent the adversary from tracing back to the location of the source node, each forwarding node determines a specific selection domain for the next-hop node according to the dangerous distance and the wireless communication range. Subsequently, it analyzes the offset angles of the candidate nodes based on the direction of the nodes to the sink node. Lastly, the forwarding node calculates the selected weights of the candidate nodes according to their offset angles. The selected weights are then used to assess which node must become the next-hop node until the packet reaches the sink node.

## 3. Models

### 3.1. Network Model

This work assumes the Panda-Hunter game network model similar to [3]. The WSN is consisted of a set of nodes and links. A wireless sensor node is a computing device equipped with a wireless interface, a limited set of computational capabilities, and has a unique identifier. Communication from a node is typically modeled with a circular communication range centered at the node. A node can exchange packets with all its neighboring nodes. A link exists between the two neighboring nodes. The network model is a large, two-dimensional coordinate network, represented by an undirected graph *G* = (*V*, *W*) with a set of vertices *V* that represent the nodes, and a set of edges *W* that represent the communication links between the neighboring nodes. All nodes are homogeneous and have the same communication range. During network initialization process, each node can locate itself, can easily learn its neighboring node locations and IDs and learn the location of the sink node. The sink node is responsible for collecting data and acts as a link between the WSN and the external world. A single sink node exists in the network.

The network is event-triggered. An event occurs when a sensor node detects an asset and becomes a source node. The source node starts sending event packets periodically to the sink node based on a multi-hop routing communication scheme. The network employs the k-nearest neighbor tracking approach [9] to track the asset. Nodes may follow a sleeping schedule, and are kept silent when no asset is detected. When a node detects the asset in its monitoring area, it remains active until the asset moves away. Packets are encrypted and contain source node ID and a timestamp to show where and when the asset is detected. Only the sink node can decode the encrypted packet. It is assumed that at any one time, only one sensor node will become a source node to avoid data redundancy and inefficient energy consumption in the network. Many sensor nodes can detect an asset but only the first node to detect the asset will become a source node and other nodes will overhear the communication from the source node and stop their communications. If multiple source nodes detect one asset at a time and each source node initiates communication with the sink, it will cause data redundancy and inefficient energy consumption. Furthermore, when multiple similar packets are reported back to the sink one at a time, it becomes easier for an adversary to trace it back to the source node. When the asset moves to a new location, it triggers another sensor node to become the new source node.

### 3.2. Adversary Model

The adversary has some technical advantages over the network sensor nodes. It is well equipped and has sufficient resources, such as adequate computation capabilities, memory and energy resources. It is equipped with antenna and spectrum analyzers, so it can observe the wireless communication within a certain detection range. The adversary is mobile, initially residing in the vicinity of the sink node listening for arriving packets. Upon detection of a packet, the adversary can measure the angle of arrival of the signal and the received signal strength to identify the immediate sender node and to perform a back tracing attack by moving to the immediate sender node location without any delay. Once at the immediate sender node, the adversary keeps on listening on the communications between the node and its neighboring nodes and continues to back trace to the source node. The adversary never misses a packet when it is within the transmission range of the receiving node.

The adversary can only perform passive attacks, such as eavesdropping the network communication, and does not interfere with the proper operation of the network. It cannot modify packets, alter the routing paths, or destroy sensor devices, since such activities can easily be detected and could put the adversary at risk of being caught. The adversary is local and cannot monitor the entire network. Kerckhoff’s principle is applied and the worst case scenario with an adversary is assumed. In the worst case scenario, it is assumed that the adversary knows the methods being used by the network. The adversary is aware of the location of the sink node, the network topology, and the routing algorithm used in the network. However, it does not know the possible location of the asset. The back tracing strategy of the adversary is summarized in Algorithm 1. The adversary is cautious and records all the sensor nodes it has visited to avoid revisiting the same nodes and getting trapped in loops. It uses a timer to limit its listening time at a node and returns to the previous node after the set timer expires.

**Algorithm 1** Adversary Algorithm1:adversaryLocation = sinkLocation2:Adversary stores the information of all visitedImmediateSenderNode in its memory3:When the adversary overhears a packet adversaryLocation = immediateSenderLocation4:At the adversaryLocation, wait for timer timeout5:**while** (adversaryLocation ! = sourceNodeLocation) **do**6:  **if** (packet comes to adversaryLocation from immediateSender before timer timeout) **then**7:   **if** (immediateSender ! = visitedImmediateSenderNode) **then**8:    adversaryLocation = immediateSender9:    Update visitedImmediateSenderNode with adversaryLocation10:   **else**11:    discard the packet12:   **end**
  **else**
13:   move to last visitedImmediateSenderNode14:  **end**15:**end**//sourceNodeLocation found

The adopted adversary model may be considered relatively weak compared to other powerful adversary models as highlighted in [16]. Nonetheless, the proposed model is preferred since some of the more powerful models include behaviors that are unlikely for the adversary to perform, as it will affect its ability to get further information from the network. Attacks, such as the denial-of-service attack, will limit the amount of useful information the adversary could gather. If the adversary tries to modify the software of the sensor node in order to collect more information about the network, the process will be time consuming and it might exceed the safety period. If the safety period is exceeded, it is possible that the asset will have moved to a different location. A set of actions that a more powerful adversary can perform but with limited benefits to the adversary are expressed in [16] in the following order: 

Eavesdrop → Crash → Disturbing → Limited passive → Passive → Reprogramming

In order to facilitate the discussions in this paper, the following terms are used:

1) Region *H* is the hotspot region near the sink node where nodes have a bigger load of packets to forward than nodes away from the sink.
2) Region *NH* is the non-hotspot region and is located far away from the sink node where nodes have a smaller load of packets to forward.
3) Diversion nodes are a set of random nodes located in region *NH*. They are used to forward packets for source nodes located in region *H.*
4) Mediate nodes are a set of random nodes located in region *NH*. They are used to forward packets for source nodes located in region *NH.*

## 4. Proposed Strategic Location-Based Random Routing Scheme

**Figure 3:**
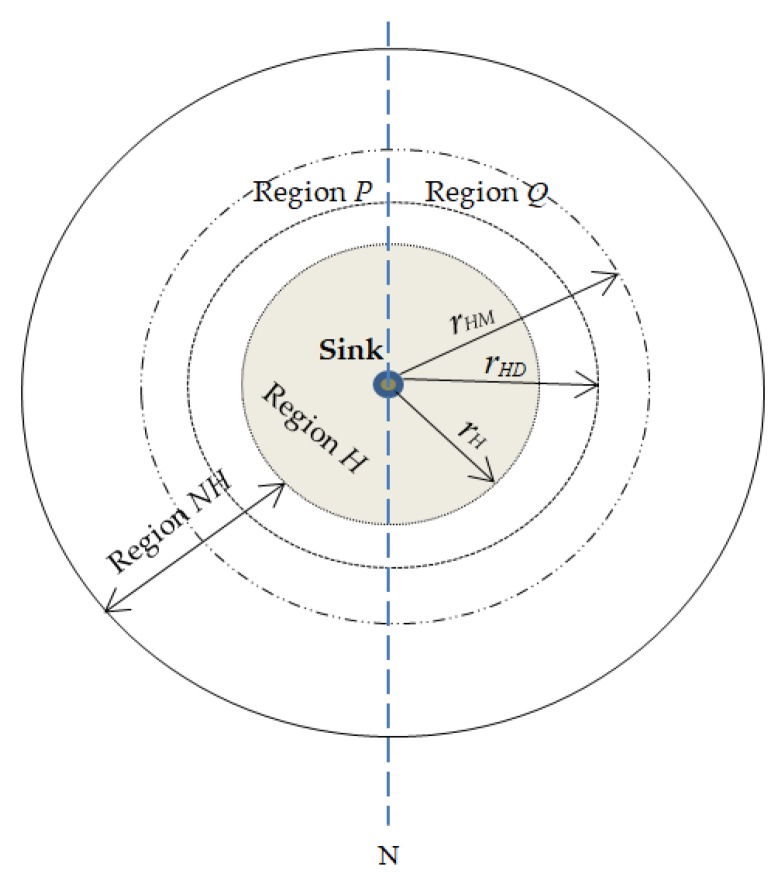
Distribution of regions in the sensor domain.

This paper proposes a location-based random routing scheme for source location privacy in WSNs. In this scheme, the sensor domain is divided into two regions: (1) hotspot region *H*; (2) non-hotspot region *NH*. Figure 3 illustrates the division of the WSN domain. *r_H_* is a pre-determined radius from the sink node to the boundary of region *H. r_HD_* is a pre-determined radius from the sink node to the outer-edge of the diversion node region. $r_{HD}-r_{H}=\mathrm{ring}\;r_{D}$. Diversion nodes are located in ring *r_D_*. *r_HM_* is a pre-determined radius from the sink node to the outer-edge of the mediate node region. $r_{HM}-r_{HD}=\;\mathrm{ring}\;r_{M}$. Mediate nodes are located in ring *r_M_.* Packets are routed in the network according to the location of the source node. Diversion nodes are strategically positioned in ring *r_D_* so as to increase the length of routing paths and control the energy consumption and delivery latency for source nodes in region *H*. If the diversion nodes are located too far away from the sink node, they will cause high delivery latency and high energy consumption. Mediate nodes are strategically positioned in ring *r_M_* to ensure that mediate nodes are far from the sink node and to make it more difficult for an adversary to back trace from sink node to the mediate node. The location of rings *r_D_* and *r_M_* guarantees that diversion and mediate nodes are positioned at minimum distance *d_min_* from the sink node. For diversion nodes, $d_{min}=r_{H}$. Distance of a diversion node from sink node is *d_D_* which will always satisfy equation $d_{D\;}>\;r_{H}$. For mediate nodes, $d_{min}=r_{HD}$. Distance of a mediate node from sink node is *d_M_* which will always satisfy equation $d_{M}>\;r_{HD}$. Distance between any two sensor nodes such as between nodes *U* and *V* is determined using the Euclidean distance equation $d_{U,V}=\sqrt{{\left(x_{U}-x_{V}\right)}^{2}+{\left(y_{U}-\;y_{V}\right)}^{2}}$. It is argued in [8] that nodes in region *H* have a bigger load of packets to forward to the sink node, hence higher energy consumption. Strategically, positioning diversion and mediate nodes in region *NH* helps distribute the energy consumption in the network.

Mediate and diversion node regions are further divided into regions *P* and *Q* according to line *N* as shown in Figure 3. Regions *P* and *Q* are 180° degrees apart with respect to the sink node. Dividing the regions into *P* and *Q* creates highly random routes with increased path variation. A source node can randomly select a route through mediate nodes in regions *P* or *Q* or through diversion nodes in regions *P* or *Q*. This ensures that routes are more random and packets will arrive at the sink node from all directions. The routes become more unpredictable to the adversary.

**Figure 4:**
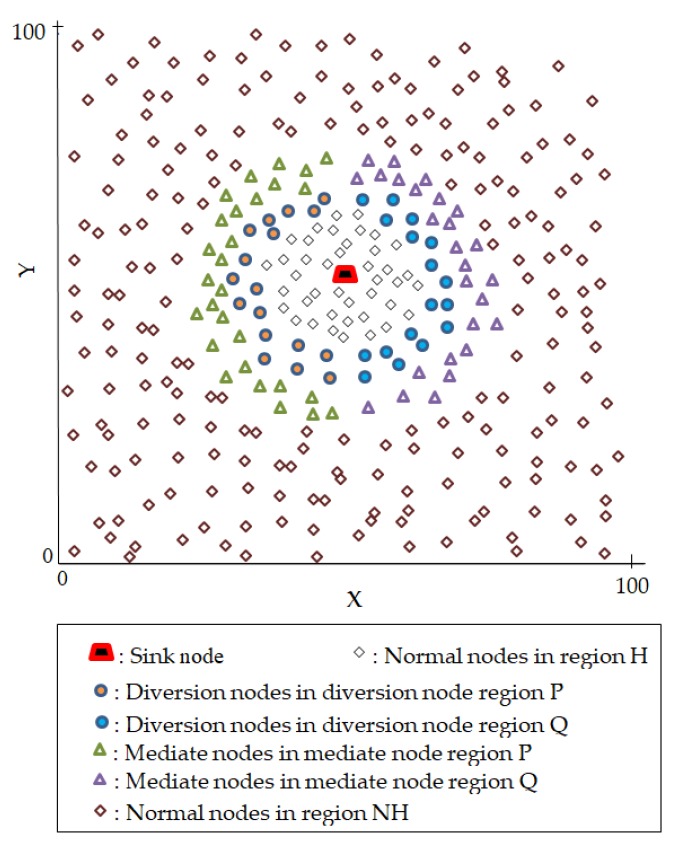
WSN domain and the strategic distribution of mediate and diversion nodes.

Figure 4 shows a WSN domain spanning 100 × 100 m² and the proposed strategic distribution of mediate and diversion nodes. A source node can be any of the nodes in regions *H* and *NH*. A source node can randomly select one mediate node or a diversion node depending on its location. All mediate or diversion nodes have equal probabilities to be randomly selected for packet forwarding. As an example, in Figure 4, a source node in region *NH* may send three consecutive packets through three very different routing paths. *Packet 1* may randomly select one mediate node from the top area of the mediate node region *P*. *Packet 2* may randomly select one mediate node from the bottom area of the mediate node region *Q*. *Packet 3* may also randomly select one mediate node from the mediate node region *Q*, but this time it selects the node from the middle area of the region. This process makes the tracing back attack of the adversary a more complicated task and therefore improves the privacy level of the proposed scheme. The operation of the proposed scheme begins with network initialization and thereafter packet routing.

### 4.1. Network Initialization

It is assumed a network operator will perform pre-deployment phase to determine the network size and division of the network according to Figure 3 and as explained in Section 4 above. Network initialization process follows after pre-deployment phase is complete. It is assumed that each node is informed about its own location, location of the neighboring nodes and of the sink node during network initialization process. The first step in network initialization process is to load each sensor node with a unique identifier (ID). Thereafter, the sink node obtains its location information using a global positioning system (GPS). The sink node then broadcasts a beacon packet to all sensor nodes in the network and sets its hop counter to zero. Each node receives the beacon packet, stores the hop counter value with a sender node ID, increments the hop counter by one, and rebroadcasts the beacon packet to its neighboring nodes. The hop counter number indicates how many hops away the sensor node is from the sink node. This gives each node information and knowledge about its neighboring nodes and its location with respect to the sink node. When a sensor node receives multiple packets, it only stores the minimum hop count in its buffer and deletes other hop counter information. After that, each sensor node calculates and records a set of its neighboring nodes. Each node informs its hop-distance to the sink. At the same time, the network operator will assign the role of the sensor nodes according to their location in the network. A sensor node is labeled as a node in the hotspot region if it is located within the radius *r_H_* and in the non-hotspot region if it is located in radius greater than *r_H_*. Parameter *r_H_* is predefined and used to determine regions *H* and *NH*. Similarly, the diversion nodes in the ring *r_D_* and the mediate nodes in the ring *r_M_* are determined and regions *P* and *Q* of the diversion and mediate nodes are determined.

### 4.2. Packet Routing 

Packet routing of the proposed scheme begins when a source node detects an asset. Upon detection of the asset, the source node generates and encrypts data packets to send to the sink node through multi-hop routing.

#### 4.2.1. Routing Strategy for Source Nodes in Region *H*

PHASE 1

Upon detection of the asset, the source node generates a bias random number *R_N_* ranging from 0 to 1 and compares it to a predefined threshold *T*. If *R_N_* is less than the threshold *T*, the source node randomly selects one of its neighboring nodes in the direction of the strategically positioned diversion node region *P*. One diversion node is randomly selected in the diversion node region *P*. Otherwise, the source node randomly selects one of its neighboring nodes in the direction of the strategically positioned diversion node region *Q*. One diversion node is randomly selected in the diversion node region *Q*. The source node determines a group of neighboring nodes with shorter hop distance to the randomly selected diversion node than the source node itself. One neighboring node from the group is randomly selected as the next-hop node. The source node randomly forwards the packet to the next-hop node and eventually to the randomly selected diversion node in regions *P* or *Q*. Phase 1 routing ends when the packet reaches the diversion node.

PHASE 2

Upon reception of the packet, diversion node determines a group of neighboring nodes with shorter hop distance to the sink node than the diversion node itself. One neighboring node from the group is randomly selected as the next-hop node. The diversion node randomly forwards the packet to the next-hop node and eventually to the destination sink node.

#### 4.2.2. Routing Strategy for Source Nodes in Region *NH*

PHASE 1

Upon detection of the asset, the source node generates a bias random number *R_N_* ranging from 0 to 1 and compares it to a predefined threshold *T*. If *R_N_* is less than the threshold *T*, the source node randomly selects one of its neighboring nodes in the direction of the mediate node region *P*. One mediate node is randomly selected from the mediate node region *P*. Otherwise, the source node randomly selects one of its neighboring nodes in the direction of the mediate node region *Q*. One mediate node is randomly selected from the mediate node region *Q*. The source node determines a group of neighboring nodes with shorter hop distance to the randomly selected mediate node than the source node itself. One neighboring node from the group is randomly selected as the next-hop node. The source node randomly forwards the packet to the next-hop node and eventually to the randomly selected mediate node in regions *P* or *Q*.

PHASE 2

Upon reception of the packet, the mediate node determines a group of neighboring nodes with shorter hop distance to the sink node than the mediate node itself. One neighboring node from the group is randomly selected as the next-hop node. The mediate node randomly forwards the packet to the next-hop node and eventually to the destination sink node. 

**Figure 5:**
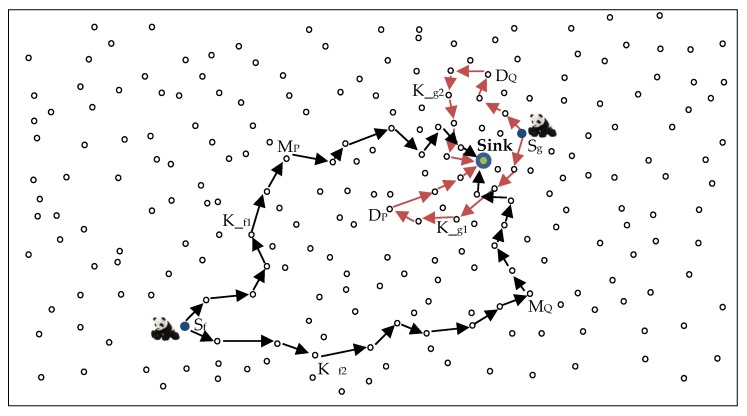
An example of a packet routing strategy using the proposed scheme.

**Figure 6:**
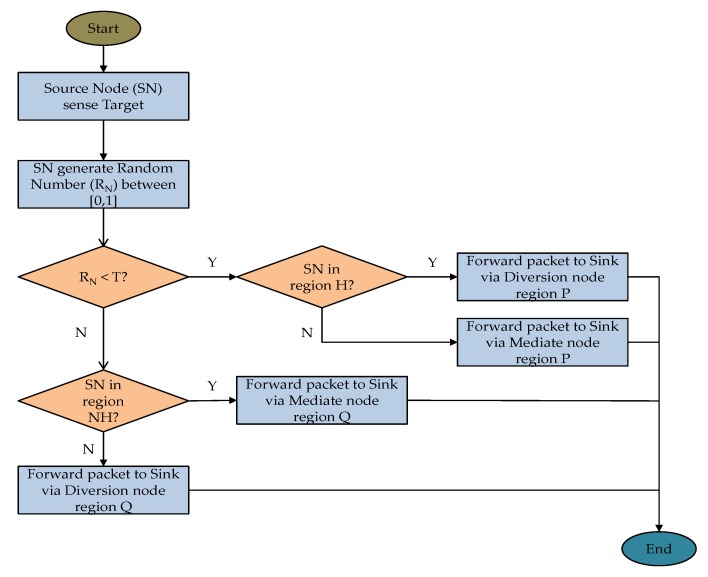
The flowchart of delivering a packet from the source node to the sink node.

Figure 5 shows an example packet routing strategy using the proposed scheme. In the figure, *S_f_* and *S_g_* are the source nodes located in regions *NH* and *H*, respectively. The figure shows that if *S_f_* sends two packets *K__f1_* and *K__f2_*, the packets will use very different route paths depending on the selected mediate node. Packet *K__f1_* uses the mediate node *M_P_* from the mediate nodes in region *P*, while *K__f2_* uses the mediate node *M_Q_* from the mediate nodes in region *Q*. Similarly, for *S_g_*, packet *K__g1_* uses the diversion node *D_P_* from the diversion nodes in region *P*, while *K__g2_* uses the diversion node *D_Q_* from the diversion nodes in region *Q*. Figure 6 shows the flowchart of delivering a packet from the source node to the sink node using the proposed routing scheme.

## 5. Privacy Analysis

To ensure the proposed scheme provides higher privacy compared to the shortest path, phantom single-path, and directional random routing schemes, the selection of the mediate node or diversion node is random and location-based. Source nodes in region *H* randomly forward packets to sink node through diversion node regions *P* or *Q*. Source nodes in region *NH* randomly forward packets to sink node through mediate node regions *P* or *Q*. The use of either the mediate or diversion node regions *P* or *Q* significantly increase the privacy level of the proposed scheme as compared to the shortest path, phantom single-path, and directional random routing schemes. As the regions *P* and *Q* are strategically positioned on opposite sides of the sink node, successive packets will use highly random routing paths and may arrive at the sink from completely different directions as shown in Figure 5. These paths will not be related as they do in the shortest path, phantom single-path, or directional random routing schemes.

Considering a cautious adversary who waits for a specified amount of time at a node and rolls back to a previous node when the timer expires, the success rate of the adversary is considerably reduced in the proposed scheme as compared to the shortest path, phantom single-path, and directional random routing schemes. Owing to the highly random routing paths in the proposed scheme, the adversary will wait for a much longer time at a node before it receives a packet at the same node again. For example, in Figure 5, if the adversary receives *K__f1_* forwarded by node *M_P_*, it will back trace to node *M_P_*. However, the next packet *K__f2_* is routed through the mediate node *M_Q_*, which is very far away from *M_P_*. If the waiting time exceeds the waiting timer, the adversary will find itself rolling back to the previous node and make insignificant progress towards the source node. For a successful back tracing attack to the source node, the adversary needs to intercept many packets. If the packets use very different routes in the network, it will take longer for the adversary to receive enough packets to intercept and successfully locate the asset. The adversary might find itself using a longer time than the safety period of the scheme and the asset will possibly move to a new location before the adversary locates the source node.

To compare the shortest path routing and the proposed scheme, if a node is located four hops away from the sink, the shortest path routing scheme will find the shortest route to the sink, which may be not more than four hops to the sink. This shortest route allows the adversary to successfully back trace to the location of the source node within a short time. Additionally, using the shortest routes increases the chances that the packets from the same source node use the same routing paths where the adversary is likely to receive successive packets and easily back trace to the source node. On the other hand, if the proposed scheme is used, when a source node is four hops away from the sink, the packets will be routed first to a random diversion node before they are randomly rerouted to the sink. This route may increase the route path from four hops to more than eight hops depending on the location of the diversion node. This difference in routing paths may also be observed in Figure 2 where the source node *A* uses the shortest path routing *E* and the proposed scheme routing path *G* to route packets to the sink node. Overall, the proposed scheme can improve the complexity for the back tracing of the source node by the adversary, and provides effective source node location privacy protection. A possible limitation of the proposed routing scheme is when a source node chooses a diversion node near its own location. This may cause a short routing path for that particular packet. However, this is not a serious limitation as there is a very high probability that the generated random number *R_N_* for the next packet will be different and the next packet will use a diversion node selected from another region causing a completely different routing path. Successive packets from the same source node are guaranteed to use completely different routes depending on the value of *R_N_*, and so adversary tracing back attacks become more difficult.

## 6. Performance Analysis and Evaluation

To evaluate the performance of the proposed scheme, simulations were carried out using MATLAB. In the simulations, the target area was a square grid network layout of size 2000 × 2000 m² with 2500 randomly distributed nodes. The sink node was the destination for all the packet transmissions and was positioned at the center of the WSN domain. The sink node was positioned at the center of the sensor domain so as to control the delivery latency in the network. The adversary was initially deployed around the sink node and it performed hop-by-hop back tracing attacks to identify the location of the source node. To control the packet delivery delay and energy consumption as explained earlier, and to ensure availability of a reliable number of diversion and mediate nodes, radius *r_H_* was set at 400 m, ring *r_D_* at 200 m, and ring *r_M_* at 200 m, following the distribution shown in Figure 3. A thorough analysis found that an optimal value of the predefined threshold *T* was 0.6 as this value guaranteed the maximum delivery ratio. Simulations were run for 400 iterations and average values were considered.

**Figure 7:**
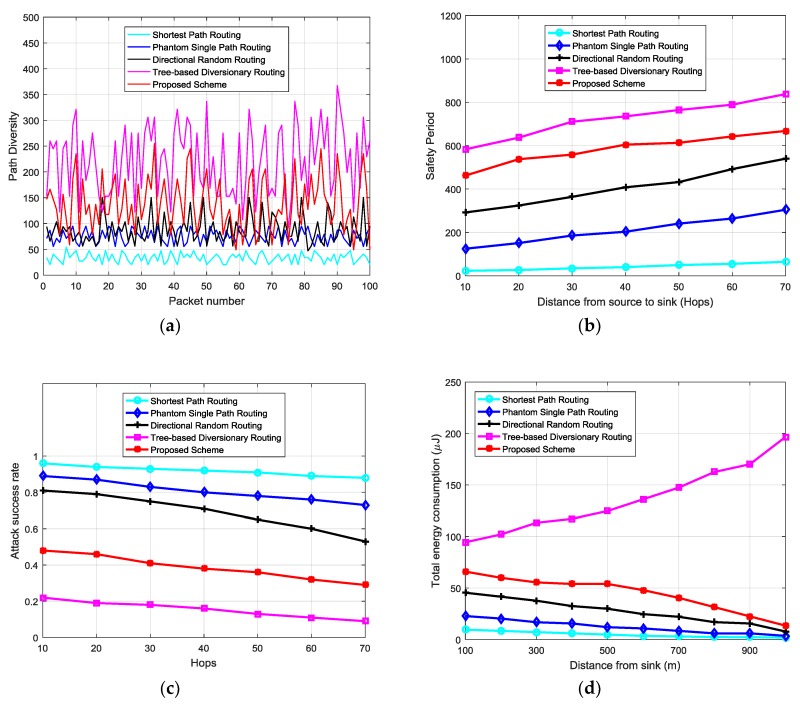

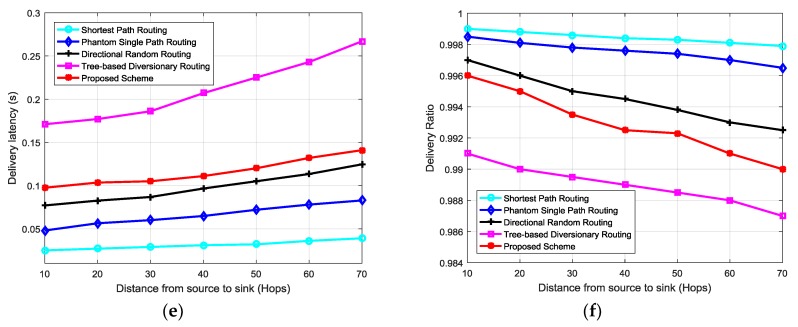
Simulation performance of proposed scheme, and for the shortest path routing, phantom single path routing, and directional random routing schemes. (**a**) Path diversity; (**b**) Safety period; (**c**) Attack success rate; (**d**) Energy consumption; (**e**) Delivery latency ; and (**f**) Delivery ratio.

**Figure 8:**
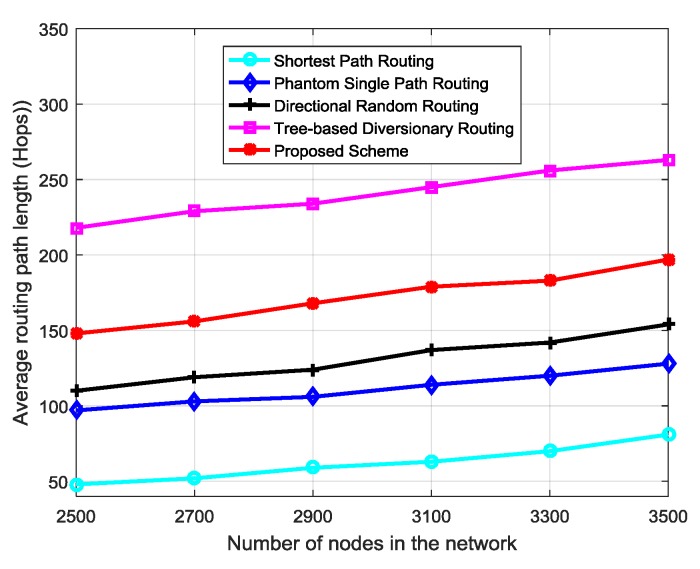
Variation of routing path length as node density increases in the network.

In the simulation, the performance of the proposed scheme was compared with that of the shortest path routing, phantom single-path routing, directional random routing and tree-based diversionary routing schemes. Tree-based diversionary routing scheme was included in the analysis because it is one of the more recent proposed schemes which provide very high source location privacy. The random walk of the phantom single-path routing scheme was set to 20% of the hops between the source and the sink nodes [9]. The network simulation parameters are summarized in Table 1. The energy consumption model is adopted from [8]. For the transmission of an *l*-bit packet by a distance *d*, the transmission energy *E_t_* and receive energy *E_r_* were defined by Equations (1) and (2), respectively [8]. The energy consumption for packet transmission was proportional to the square of the transmission distance *d*. *E_elec_* denotes transmitting circuit loss. The energy consumption model uses both, the free space (*d*^2^ power loss) and the multi-path fading (*d*^4^ power loss) channel models. If the transmission distance is less than the threshold *d*_0_, the power amplifier loss is based on free-space model. If the transmission distance is equal or greater than the threshold *d*_0_, the multi-path attenuation model is used. *E_fs_* and *E_amp_* are the energy required by power amplification in the two models. Simulation results in Figure 7 and Figure 8 demonstrate the performance of the schemes. Performance metrics include the path diversity, safety period, attack success rate, delivery latency , and energy consumption, and are used for further analyses:

(1) $$\left\{\begin{array}{c} E_{t}=lE_{elec}+lE_{fs}d^{2},\;if\;d\;<\;d_{0}\\ E_{t}=lE_{elec}+lE_{amp}d^{4},\;if\;d\;\geq \;d_{0} \end{array}\;\right\}$$

(2) $$E_{r}=lE_{elec}$$

Figure 7a shows the path diversity of the analyzed schemes. Privacy is directly related to path diversity. As the path diversity available for each packet is enhanced, higher source location privacy is guaranteed. Path diversity can be categorized into length and path variations. 

In reference to the length variation, the source node can forward packets to the sink using paths with different lengths at each time it forwards a packet. Longer paths increase the safety period and privacy, and vice versa. In reference to path variations, each individual packet follows a different route to the sink. Higher path variation improves the privacy by making it more difficult for an adversary to guess which route the next packet will use. The figure shows the path diversity in terms of the path length for a single source node which generates and forwards 100 packets to the sink. It shows that the proposed scheme has a much higher path diversity than the shortest path routing, phantom single-path routing and directional random routing schemes. The tree-based diversionary routing scheme has a higher path diversity than the proposed scheme because it integrates many routing techniques. It creates backbone routes which are directed to the network border with many diversionary routes as the branches. Packet routes are directly routed to the network edge from the sink node. At the end of each diversionary route, the scheme generates fake source nodes which emits fake packets periodically to lead the adversary away from the real packet route. The scheme also uses phantom nodes located far away from the source node. Combination of all these routing techniques provides a much higher path diversity. The average path diversity for the proposed scheme is 148 hops, while the shortest path routing has 42 hops, the phantom single-path routing has 92 hops, the directional random routing has 108 hops, and the tree-based diversionary routing has 211 hops. The high path diversity in the proposed scheme is achieved by the longer and more random routing paths, which go through either mediate node or diversion node regions. 

A parameter called safety period is used to measure source location privacy in WSNs. Two notions for the parameter safety period are given in [16]. The first notion defines the safety period as the time required for an adversary to back trace and capture the asset. The second notion defines the safety period as the maximum time an asset will be at a given location before its next movement. Longer safety periods provide higher privacy levels. Figure 7b shows the safety period of the analyzed schemes. The proposed scheme has a much higher safety period than the shortest path routing, phantom single-path routing and directional random routing schemes while the tree-based diversionary routing scheme has the highest safety period. The safety period for all the schemes increases as the distance from the source node to the sink node increase. For source nodes near the sink, the proposed scheme provides longer safety period than shortest path routing, phantom single-path routing, and directional random routing schemes. Overall, the proposed scheme has a good safety period and privacy performance, especially for source nodes near the sink due to its highly random routing paths. The tree-based diversionary routing scheme has higher safety period, but at a cost of implementing a combination of many routing techniques which may lead to costs such as higher energy consumption and higher delivery latency. As the asset is assumed to be mobile, if the adversary performs an exhaustive search of the network for a back tracing attack, the proposed scheme will guarantee a long safety period. With the long safety period, it could become possible that the asset will have moved to a different location by the time the adversary located the source node.

Attack success rate measures the rate of source traceability when using a routing scheme against the back tracing adversary. It is determined by counting the number of successful adversary attempts. A longer safety period for a scheme will reduce the success rate of the adversary. Figure 7c shows the attack success rate of the proposed scheme as compared to the other schemes. The proposed scheme has a low attack success rate because it uses highly random routes between successive packets. The tree-based diversionary routing scheme has the lowest attack success rate because it is more confusing for adversary to back trace the long routing paths which divert to the network border and involve fake packet sources, many diversionary routes and phantom nodes. The shortest path routing has the highest attack success rate because it delivers packets through the shortest paths, which are easy for the adversary to back trace. Phantom single-path routing outperforms the shortest path routing because it employs random walk routing which improves its performance. If the adversary attacks the proposed scheme at a distance of 20 hops, it has a success rate of 46%, but if it attacks the other schemes, it has 79% success rate for directional random routing, 87% success rate for phantom single-path routing, 94% for the shortest path routing, and 19% for the tree-based diversionary routing.

The proposed scheme provides high level of source location privacy at slightly higher transmission costs as compared to shortest path routing, phantom single-path routing and directional random routing schemes. However, the costs are lower than the costs in tree-based diversionary routing scheme. Figure 7d–f show the energy consumption, delivery latency and delivery ratio performances respectively. Comparing Figure 7b–d, it is observable that the proposed routing scheme provides higher safety period and reduced attack success rate at a cost of higher energy consumption than shortest path routing, phantom single-path routing and directional random routing schemes. The high energy consumption in the proposed scheme is caused by the longer and highly random routing paths. Energy consumption is always optimal when packets are routed from the source node to the sink through the shortest path. The shortest path routing scheme has lowest energy consumption and lowest safety period because it delivers packets to sink via the shortest path. Directional random routing and phantom single-path routing schemes have higher energy consumption than shortest path routing because they employ longer random routes between source and sink nodes. The tree-based diversionary routing scheme has the highest energy consumption because each routing path is diverted to the network border from the sink node with many diversionary routes which consume a lot of energy. The scheme also generates fake packets which increases the energy consumption in the network. The total energy consumption of the proposed scheme, shortest path routing, phantom single-path routing and directional random routing schemes is higher near the sink node. This is owing to the bigger load of packets being forwarded to sink in the near-sink region as compared to regions away from the sink. The tree-based diversionary routing scheme has higher energy consumption in regions away from the sink node due to the usage of diversionary routes which divert the packets to the network border regions before sending them to the sink node. The use of fake packet sources at the end of the diversionary routes increases the energy consumption in network border regions. Overall, the proposed routing scheme can provide high source location privacy protection than the shortest path routing, phantom single-path routing and directional random routing schemes. However, the longer and highly random routing paths of the proposed scheme cause longer delivery latency and reduced delivery ratio as shown in Figure 7e,f. A lower delivery ratio is owing to the increased collisions and packet losses. The tree-based diversionary routing scheme has the highest delivery latency and lowest delivery ratio. The highest delivery latency is caused by the very long routing paths which divert to the network border. Lowest delivery ratio is caused by the long routing paths and the use of fake packets which increase the probability of packet collisions. The energy consumption of the proposed scheme can be improved by a more strategic distribution of mediate and diversion nodes in the network. Also, the angle-based routing technique used in [15] can be applied to reduce the energy consumption of the proposed scheme. In [15], rectangular coordinate technique is used in the process of next-hop selection to minimize the lengths of routing paths and nodes with smaller offset angles are given higher priority to be selected as next-hop nodes. The proposed scheme is good for applications which require high source location privacy but with relaxed requirements in delivery latency, delivery ratio, and tolerance of slightly higher energy consumption.

Privacy performance of a routing scheme increases as the length of routing paths (hops) between source node and sink node increase [8]. When routing paths are longer (more hops), it takes longer for an adversary to back trace and locate the source node. As number of nodes in the network increase, length of routing paths (hops) between source and sink nodes may increase. With the use of random *R_N_* for each packet in the proposed scheme, the longer routes become random and more unpredictable to the adversary. Figure 8 shows performance of the routing schemes as number of sensor nodes in the network increase. The path length of all schemes increases as the number of nodes increase. Since privacy level is proportional to the routing path length [8], it can be perceived that source location privacy of the routing schemes increases with increase in node density. Similarly, increased number of neighboring nodes and nodes in regions *r_M_* and *r_D_* of the proposed scheme will increase the path diversity between successive packets. For example, assume a source node *S_3_* has *n* neighboring nodes, probability of selecting a particular neighboring node is 1/n. When the random number *R_N_* is applied during selection of neighboring node, the selection becomes random. If region *r_D_* has *u* diversion nodes, the probability of *S_3_* selecting a particular diversion node in region *r_D_* is 1/u. Then, the total possible number of routing paths between *S_3_* and the sink node is nu. This shows that, number of routing paths is proportional to number of neighboring nodes and nodes in regions *r_D_* and *r_M_*. The improved path diversity between successive packets will make the routes more confusing to the adversary and improve privacy level.

## 7. Conclusions and Future Work

WSNs require secure routing schemes to prevent adversaries from finding out the source node location by back tracing attacks. To meet these requirements, this paper has proposed a location based random routing scheme. The scheme divides the WSN domain into two regions and packets are randomly forwarded to the sink node through random diversion or mediate nodes according to the location of the source node. The diversion and mediate nodes are strategically positioned in the sensor domain to ensure that packets for source nodes near the sink node are routed through longer and more random routes as compared to other schemes. If the adversary decides to perform a back tracing attack against the proposed scheme, it will certainly be confused in the effort to guess the route for the next packet. A cautious adversary will achieve very little progress towards the source node since the routing paths have high path diversity and successive packets from the same source node use highly random routes. Simulation results show that the proposed scheme can provide strong source location privacy and outperform traditional routing schemes, even when a source node is located in near-sink regions. The proposed scheme is applicable in systems which require high source location privacy and can tolerate an acceptable increase in energy consumption and communication overhead. As part of future work, techniques to improve energy consumption in the network will be considered.

## Figures and Tables

**Table 1:** Network simulation parameters.

Parameter	Value
Network size (m^2^)	2000 × 2000
Number of nodes	2500
Sensor node sensing range (m)	30
Adversary sensing range (m)	30
Target monitoring scheme	k-nearest neighbor tracking
Initial energy (J)	0.5
Threshold distance (d_0_) (m)	87
*E_elec_* (nJ/bit)	50
*E_amp_* (pJ/bit/m^4^)	0.0013
*E_fs_* (pJ/bit/m^2^)	10
Packet length (bit)	1024
